# The dual axis of tumorigenesis: MAPK and PI3K/AKT pathways in papillary thyroid carcinoma

**DOI:** 10.18632/oncoscience.663

**Published:** 2026-06-03

**Authors:** Gunvanti Rathod, Pragnesh Parmar

**Affiliations:** ^1^Department of Pathology, AIIMS, Bibinagar, Telangana 508126, India; ^2^Department of FMT, AIIMS, Bibinagar, Telangana 508126, India

**Keywords:** papillary thyroid carcinoma, MAPK pathway, PI3K/AKT pathway, RET/PTC, targeted therapy

## Abstract

The MAPK and PI3K/AKT pathways are important components in cell growth and proliferation, which, when activated, result in cancer formation. Molecular alterations are the main cause of activation of the MAPK and PI3K/AKT pathways in the development of papillary thyroid carcinoma (PTC), the most common endocrine cancer. Activation of the MAPK pathway is usually caused by mutations in the BRAF, RAS, RET/PTC, and NTRK genes, while the activation of the PI3K/AKT pathway results from alterations in the PIK3CA, PTEN, and AKT genes. Moreover, hyperactivation of the MAPK pathway can be caused by overexpression of RTKs, GPCRs, and mutation in PTEN. Interactions between the two signaling pathways exacerbate tumor aggressiveness, dedifferentiation, radioiodine resistance, and response to targeted therapy in PTC. Recent developments in personalized medicine, including the introduction of new molecular diagnostic tools and targeted agents, have made considerable progress in the risk stratification and treatment strategies for papillary thyroid carcinoma. The current article reviews molecular mechanisms of activation of MAPK and PI3K/AKT pathways, their interaction, clinicopathological importance, and targeted treatments in papillary thyroid carcinoma.

## INTRODUCTION

Thyroid carcinoma is the most prevalent endocrine malignancy in the world, wherein papillary thyroid carcinoma (PTC) constitutes about 80–85% of all thyroid tumors. Based on recent data obtained from GLOBOCAN 2022, thyroid carcinoma stands out as one of the malignancies with the highest incidence in the world, with an estimated number of 820,000 yearly incidences and a distinct preponderance of female patients due to various diagnostic factors and environmental exposures [1]. The vast majority of PTCs have a benign and relatively favorable course with high survival rates; however, a substantial fraction of PTCs presents with aggressive characteristics such as extra-thyroidal invasion, nodal involvement, metastases, recurrence, de-differentiation, and RAI resistance [1, 2]. During the last 20 years, remarkable progress in the field of molecular pathology has led to an enhanced comprehension of the mechanisms involved in the etiology of thyroid carcinomas. The initiation and progression of papillary thyroid cancer involve an abnormal functioning of intracellular signaling pathways responsible for regulating proliferation, differentiation, cell survival, metabolism, and apoptosis. Within such signaling pathways, MAPK and PI3K/AKT signaling pathways stand out as the main two oncogenic signal transduction cascades responsible for the pathogenesis of thyroid tumors. Such cascades can become deregulated due to various causes [3].

The MAPK pathway has been found to be mainly activated by mutations and translocations such as BRAF, RAS genes, and RET/PTC/NTRK gene fusions. However, of these mechanisms of MAPK pathway activation, BRAF V600E mutation is the commonest molecular change in PTC, with high clinical-pathologic aggressiveness, poor iodine utilization, and low response to therapy. Activation of the PI3K/AKT pathway is mainly found to occur during tumor progression and dedifferentiation processes, with mutations in PIK3CA, AKT, and PTEN genes [4]. Crucially, receptor tyrosine kinase (RTK) mediated signaling via receptors such as RET, EGFR, VEGFR, FGFR, PDGFR, as well as GPCR mediated pathways, have been reported to activate both MAPK and PI3K/AKT pathways. Additional findings have shown that there is substantial crosstalk between these two pathways. This crosstalk facilitates cancer progression, metabolic switching, epithelial-mesenchymal transition, angiogenesis, immune escape, and drug resistance. The activation of alternative pathways after the specific inhibition of one pathway has been observed as an important cause of drug resistance in advanced and RAI refractory PTC [5]. The application of molecular diagnostics in assessing and stratifying patients with thyroid nodules has considerably increased the accuracy of diagnosis and personalized therapy. In addition, the advent of molecularly targeted drugs targeting BRAF, RET, NTRK, MEK, and PI3K/AKT/mTOR pathway elements has transformed the therapeutic approach for advanced thyroid cancers [6].

This review focuses on the molecular basis of MAPK and PI3K/AKT pathway activation in PTC, clinical significance, pathway crosstalk, and mechanisms of resistance, as well as the emerging role of targeted agents in precision oncology.

### MAPK Pathway activation in papillary thyroid carcinoma

The signaling pathway mediated by the mitogen-activated protein kinase (MAPK) plays an important role in the development and progression of PTC. Normally, this signaling pathway controls cell proliferation, cell differentiation, apoptosis, and survival via RTKs, RAS proteins, RAF kinases, MEK, and extracellular signal-regulated kinase (ERK). In PTC, the MAPK signaling pathway is activated continuously due to the presence of mutations, chromosomal translocations, increased expression of receptors, and inappropriate activation of the pathway, which leads to uncontrolled proliferation of cells [2, 7].

All types of genetic alterations in the MAPK pathway are common, but the BRAF V600E alteration is the most important one among all, being found in 40-60% of PTC patients. This alteration is caused by transition from thymine to adenine at nucleotide 1799, causing amino acid substitution of valine to glutamic acid at codon 600. In this case, the BRAF kinase acquires constant kinase activity without requiring any stimulation by the receptor, which, in turn, causes activation of MEK and ERK continuously. This mutation activates ERK signaling significantly stronger than other mutations in the MAPK pathway [4, 6]. On the molecular level, the continuous signaling from ERK leads to the production of genes encoding proliferation, angiogenesis, metastasis, and anti-apoptosis, such as cyclin D1, MYC, VEGF, and anti-apoptotic proteins. At the same time, the presence of BRAF V600E mutation results in decreased expression of thyroid-specific genes, including sodium/iodide symporter (SLC5A5), thyroglobulin, and thyroid peroxidase [7, 8]. The lack of differentiation results in decreased iodine uptake and the development of dedifferentiation associated with increased insensitivity to RAI treatment. It should be noted that the decreased expression of NIS gene is critical not only for poor efficacy of RAI treatment but also because of possible complications during radioiodine imaging due to decreased sensitivity [9]. Clinically, the BRAF V600E mutation is associated with the classical and tall cell types of PTC and shows a correlation with extrathyroidal growth, nodal metastasis, recurrence, advanced disease stage, and poor disease-free survival rate. Additionally, co-presence of the BRAF V600E mutation along with TERT promoter mutations predicts more aggressive tumor behavior including high recurrence rates, distant metastases, dedifferentiation, and disease-related death. As a result, BRAF V600E is an increasingly valuable molecular marker for use in contemporary staging and treatment decisions [3, 8].

The second major mechanism of MAPK pathway activation in PTC involves RET/PTC gene rearrangements. These gene rearrangements occur due to chromosomal translocations or inversions that involve the proto-oncogene RET, resulting in the formation of fusion proteins that possess active tyrosine kinases. Commonly found RET/PTC gene rearrangements include RET/PTC1 (CCDC6::RET) and RET/PTC3 (NCOA4::RET). Activated RET causes uncontrolled signaling through the MAPK pathway without ligand binding as well as inflammation and cytokine signaling. RET/PTC gene rearrangements are commonly found in irradiated and pediatric PTCs [9, 10]. RET/PTC-positive cancers often occur with multifocal presentation and have nodal metastasis, but unlike BRAF-mutated tumors, they usually retain their thyroid differentiation and response to RAI treatment, providing a relatively better prognosis. The activation of RAS family members, such as HRAS, NRAS, and KRAS, is another important group of MAPK alterations found in thyroid cancer. Being positioned upstream from both MAPK and PI3K/AKT pathways, RAS protein molecules operate as molecular switches controlling intracellular signaling cascades. The most common RAS activating mutations are located in codons 12, 13, and 61, producing constitutively active GTP-bound forms of RAS molecules [11, 12].

As opposed to BRAF V600E mutation, RAS alterations provide a relatively weaker MAPK signaling pathway activity while also promoting PI3K/AKT signaling. As a result, RAS-mutated cancers usually maintain follicular architecture and have retained thyrocyte differentiation, accounting for their relatively benign clinical course. Such alterations are typically seen in follicular-patterned cancers, including follicular variant PTC and NIFTP. Clinically, RAS-mutated cancers demonstrate lower prevalence of nodal metastasis and relatively good prognosis [5, 11]. Another example of oncogenic driver mutations includes NTRK1/3 and ALK gene fusions. They produce fusion kinases that can activate MAPK signaling. Being rare events, they are clinically significant due to the presence of highly targetable kinase inhibitors. Tumors that have a NTRK fusion tend to show an invasive growth pattern and a capacity for distant spread, although these neoplasms are highly sensitive to TRK kinase inhibitors [12, 13].

In addition to genetic alterations and chromosomal rearrangements, there is another mechanism for activating the MAPK pathway that should be mentioned. Abnormal activation of the upstream receptors, including RTKs and GPCRs, leads to enhanced activity of the RAF/MEK/ERK cascade. Excessive activation and overexpression of RTKs, such as EGFR, VEGFR, FGFR, PDGFR, MET, and RET, amplify the activity of the downstream signaling cascade. Similarly, abnormal signaling of GPCR receptors can increase the phosphorylation of ERK [14, 15]. Thus, pathway activation can be considered as the main molecular driver of papillary thyroid cancer development. There is some heterogeneity in the biological effects caused by pathway activation that depend on the exact molecular mutation. In turn, it determines differentiation grade, aggressiveness, metastatic potential, sensitivity to radioiodine therapy, and vulnerability to targeted drugs.

### PI3K/AKT pathway activation in papillary thyroid carcinoma

PI3K/AKT pathway is responsible for regulation of cellular metabolism, proliferation, growth, survival, angiogenesis, and apoptosis. While MAPK pathway activation is dominant in the early development of papillary thyroid carcinoma (PTC), defects in the PI3K/AKT pathway make significant contributions to cancer progression, dedifferentiation, metastasis, and chemoresistance [13, 14]. There are several ways of PI3K/AKT pathway activation, which include mutations of PIK3CA and AKT genes, PTEN loss, RTK overexpression, and activation via GPCRs. Physiologically, binding of ligands to RTKs, such as EGFR, VEGFR, FGFR, PDGFR, MET, and RET activates PI3K, resulting in PIP2 to PIP3 transformation. This reaction leads to PI3K recruitment and activation, followed by phosphorylation of downstream effectors, which promote protein synthesis, cell cycle progression, glucose metabolism, and apoptosis inhibition. Chronic stimulation of this pathway results in increased cell survival and cancer development [15, 16].

PIK3CA mutation leads to the continuous activation of PI3K, leading to activation of AKT signaling. However, it should be noted that despite their low incidence in well-differentiated PTC, mutations of PIK3CA are more common in poorly differentiated thyroid carcinoma and anaplastic thyroid carcinoma, implying that they may act as secondary genetic abnormalities. Continuous PI3K signaling is associated with cell proliferation, metabolic rewiring, angiogenesis, and cell resistance to apoptosis, thus leading to malignant cell behavior [9, 17]. From a transcriptional and cellular standpoint, continuous PI3K/AKT activation increases mTOR signaling, thus leading to cell growth and metabolic changes. On the other hand, AKT activation leads to the inhibition of pro-apoptotic molecules like BAD, as well as inhibition of the expression of transcription factors FOXOs [18].

One of the most important ways of activating the PI3K/AKT pathway in thyroid cancer is through PTEN dysfunction. PTEN is a tumor suppressor gene whose function is to inhibit PI3K pathway activation by converting PIP3 back to PIP2, thus preventing AKT phosphorylation. PTEN loss, which can be brought about by mutations, deletions, or silencing, will result in constitutive and unregulated PI3K/AKT pathway activation. PTEN dysfunction has been shown to lead to tumor aggressiveness, local invasiveness, metastasis, de-differentiation, and resistance to treatment [19, 20]. Moreover, PTEN loss will enhance resistance to treatment by ensuring cell survival even when MAPK inhibitors are used. Consistent AKT activation will help tumor cells resist MAPK inhibition and thus prevent cell death. It is now widely known that such adaptive mechanisms play an important role in resistance to BRAF and MEK inhibitors in advanced thyroid cancer cases [21]. Moreover, overexpression of the AKT kinases, namely AKT1, AKT2, and AKT3, was identified in highly aggressive forms of thyroid cancer. Despite being relatively rare in classic PTC, AKT overactivation is known to promote increased proliferation and invasion, as well as resistance to the effects of RAI treatment. AKT hyperactivity inhibits the expression of thyro-differentiation-related genes, such as NIS, which interferes with iodine accumulation and induces radioiodine resistance in cancer cells. Thus, this mutation may result in decreased radioisotope uptake in diagnostics [20, 22]. Apart from mutations, hyperactivation of the RTK and GPCR signaling may substantially contribute to the increased activity of the PI3K-AKT pathway. Excessive expression of RTKs, such as EGFR, VEGFR, PDGFR, FGFR, MET, and RET, promotes the simultaneous activation of the PI3K/AKT and MAPK pathways. Moreover, hyperactivation of the signaling pathway mediated by intracellular G proteins activated via GPCR receptors may result in PI3K over activity, either directly or indirectly [23, 24].

In terms of the clinical relevance, the PI3K/AKT pathway alteration is more commonly seen in aggressive and dedifferentiated thyroid cancer, rather than in early-stage conventional PTC. Mutations in the PIK3CA gene and PTEN deletions are usually associated with high-grade tumors, invasive potential, distant metastases, recurrence, and unfavorable outcomes. These genetic events are most prevalent in poorly differentiated thyroid carcinoma and anaplastic thyroid carcinoma [25, 26]. The PI3K/AKT pathway is important for the development of resistance to the therapy. The blockage of the MAPK pathway may trigger compensatory activation of the PI3K/AKT pathway due to receptor tyrosine kinase upregulation and feedback activation. On the contrary, inhibition of PI3K leads to activation of the MAPK pathway via breaking the negative feedback loop. This two-way compensatory signaling pathway supports the survival of tumor cells and decreases the effectiveness of single-agent treatment [27, 28]. Understanding of the complex interplay between molecules has stimulated research in dual targeted therapy. Numerous investigational drugs against the PI3K, AKT, and mTOR pathways have been studied for advanced thyroid cancers resistant to traditional treatments and MAPK inhibitors [3, 15].

In summary, the activation of the PI3K/AKT pathway is an important molecular step involved in the development of thyroid cancer, thyroid cancer de-differentiation, and resistance to therapy. The collaboration between the MAPK and PI3K/AKT pathways enhances the oncogenic potential of this phenomenon and helps to explain the biological heterogeneity seen in papillary thyroid carcinoma. It is important to understand these mechanisms for developing targeted therapies against thyroid cancer.

### The molecular cross talk between MAPK and PI3K/AKT signaling pathways

Although MAPK and PI3K/AKT signaling pathways have been viewed separately as independent signaling pathways, recent evidence suggests that there is considerable cross-talk at the molecular level between these two pathways in papillary thyroid carcinoma (PTC). Both pathways regulate similar physiological phenomena in cell biology, such as growth, survival, metabolism, differentiation, angiogenesis, migration, and apoptosis [29, 30]. Interaction between these pathways takes place at many different levels that include receptor activation, oncogene mutation, adaptors, and feedback loops. At the cell surface level, RTKs such as RET, EGFR, VEGFR, FGFR, PDGFR, MET, and IGF-1R can simultaneously activate both MAPK and PI3K/AKT pathways [31, 32]. Activation of these receptors by ligands leads to their dimerization and subsequent autophosphorylation, allowing the recruitment of adaptors like GRB2, SOS, SHC, and PI3K. The activation of GRB2/SOS leads to the RAS-Raf-MEK-ERK pathway, whereas the activation of PI3K leads to AKT signaling via PIP3 production.

Additionally, G protein-coupled receptors (GPCRs) also play a role in simultaneous activation of the pathways. GPCR activation results in the activation of G proteins inside the cells, which can trigger ERK phosphorylation and PI3K activation through intermediary kinases and second messengers. This type of receptor-dependent signaling serves to enhance oncogenic pathway activation, especially for late-stage or resistant tumors [34, 35]. RAS proteins play a special role in pathway crosstalk due to being located upstream of both signaling pathways. Mutations in any of HRAS, NRAS, or KRAS will cause the activation of both RAF/MEK/ERK and PI3K/AKT pathways, leading to synchronized cell proliferation and survival. While mutations such as BRAF V600E activate MAPK signaling exclusively, RAS mutations result in dual activation of both signaling pathways; this difference accounts for the biological and clinical behavior of each type of mutation [23, 36].

Further crosstalk happens on a level of downstream signals. Continuous activation of ERK might lead to enhanced mTOR signaling and metabolic adaptation, while active AKT could impact RAF and ERK through the modulation of intermediate signaling molecules. As such, mTOR becomes an important molecule mediating the connections between growth factor signaling, protein translation, and nutrient sensing [30, 35].

The most important example of molecular interaction in PTC is the presence of both BRAF V600E and TERT promoter mutations in the same cancerous cell. Mutation in BRAF causes persistent ERK activation that results in increased transcription from TERT promoter mutations by binding of ETS transcription factors. This synergistic mechanism enhances telomerase activity, leads to immortalization, promotes genomic instability, tumor growth, recurrence, and disease-specific mortality rates [23, 29]. In relation to transcription, MAPK and PI3K/AKT activation in tandem leads to increased transcription of genes involved in cell growth, angiogenesis, invasion, and epithelial-mesenchymal transition while reducing the expression of differentiation markers of thyroid tissue. Persistent activation of these signaling molecules results in decreased expression of the gene for sodium-iodide symporter (NIS), thyroglobulin, and thyroid peroxidase, leading to reduced ability of uptake of iodine and increased resistance to treatment with radioactive iodine [36, 37].

Pathway cross-talk is an important mechanism responsible for therapeutic resistance, and selective inhibition of a single pathway often leads to activation of another pathway by compensatory means. Specifically, the use of drugs that block MAPK, such as BRAF inhibitors for BRAF V600E mutations, could lead to upregulation of receptor tyrosine kinases like EGFR, HER2, and MET and subsequent activation of PI3K/AKT pathway. Inhibition of MEK also relieves negative regulation of RTKs and Ras, resulting in reactivation of parallel survival pathways [34]. Similarly, targeting PI3K and mTOR pathways can lead to activation of ERK pathway by relieving the negative regulation of receptor tyrosine kinases and Ras proteins. The crosstalk between the MAPK and PI3K/AKT pathways further impacts the tumor microenvironment. The cooperation results in promoting angiogenesis via VEGF induction, immunosuppression, ECM remodeling, and metastasis. In poorly differentiated and anaplastic thyroid carcinomas, coactivation of both pathways occurs commonly [23, 25].

Clinically, malignancies showing coactivation of the two pathways show aggressive histology, high recurrence rate, metastasis to distant sites, decreased response to radioactive iodine treatment, and poor prognosis. With the awareness of such molecular interactions, there has been increasing interest in therapies that target both pathways at the same time [38]. Several preclinical and early-stage clinical trials have indicated that concomitant blocking of the MAPK and PI3K/AKT/mTOR pathways can exhibit synergism in suppressing malignancy, overcoming resistance, inducing thyroid differentiation, and enhancing radioactive iodine uptake. Therefore, combination treatments such as BRAF inhibitors, MEK inhibitors, PI3K inhibitors, AKT inhibitors, and mTOR inhibitors are promising therapies against advanced and refractory thyroid cancers [2, 34]. On the whole, the significant amount of inter-pathway molecular crosstalk between the MAPK and PI3K/AKT signaling pathways constitutes a crucial element responsible for tumor progression, dedifferentiation, and therapy resistance observed in papillary thyroid carcinoma. Knowledge regarding molecular pathways involved in papillary thyroid carcinoma is key to enhancing molecular risk stratification and creating precision-based therapies for treatment in advanced thyroid cancer patients.

**Figure 1:**
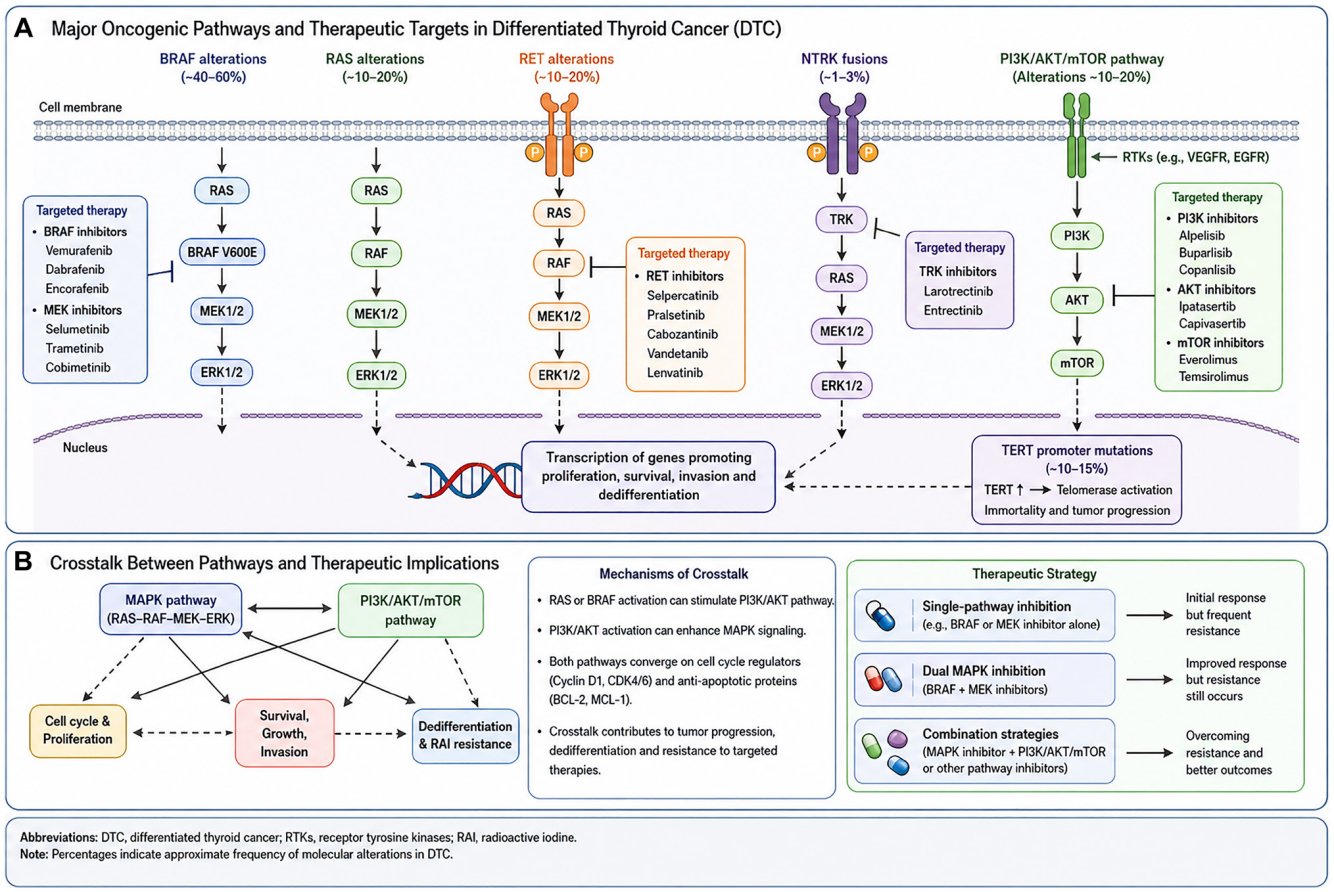
Dual oncogenic activation and molecular crosstalk of MAPK and PI3K/AKT pathways in papillary thyroid carcinoma. (**A**) representing the activation of MAPK and PI3K/AKT pathways through oncogenic mutations (BRAF, RAS, RET/PTC, NTRK), RTK overexpression, GPCR signaling, and PTEN deficiency. (**B**) Cross-talk between pathways is facilitated by common upstream activators, RAS signaling, mTOR integration, and feedback signaling. Compensation for activation in resistant cancers is shown.

The mechanisms of MAPK and PI3K/AKT pathway activation, including upstream receptor signaling, genetic alterations, and molecular crosstalk contributing to therapeutic resistance, are illustrated in Figure 1.

### Molecular testing in thyroid nodules

Introduction of molecular diagnostics into the clinical picture of thyroid nodules is one of the greatest achievements in managing papillary thyroid carcinoma. While fine-needle aspiration cytology (FNAC) remains a standard test method for thyroid nodules, nearly 15–30% of nodules will be deemed indeterminate based on the Bethesda System for Reporting Thyroid Cytopathology. Particularly, Bethesda categories III (Atypia of undetermined significance/follicular lesion of undetermined significance) and IV (follicular neoplasm/suspicious for follicular neoplasm) usually refer to those nodules that are indeterminate. Previously, these indeterminate lesions needed diagnostic surgery since cytomorphology was not enough for differentiating between benign and malignant follicular-patterned lesions [30, 35]. Recent developments in molecular pathology have made it possible to detect genetic alterations responsible for thyroid carcinogenesis, enhancing diagnostic precision, evaluation of malignancy risk, prognosis, and treatment options. Currently, molecular analysis is becoming indispensable in individualized thyroid nodule and differentiated thyroid carcinoma management [12, 23].

Among all detected molecular alterations in PTCs, BRAF V600E stands out as the most clinically informative. BRAF V600E shows excellent specificity to classical and tall cell variants of PTC. This mutation is virtually absent in benign thyroid nodules. Detection of BRAF V600E in FNAC samples increases the likelihood of diagnosing cancer and suggests a more aggressive biological behavior characterized by extrathyroidal growth, regional lymph node involvement, recurrence, and lower radioiodine uptake. In addition, it predicts the inhibition of thyroid differentiation factors, e.g., NIS, impacting both treatment and imaging outcomes [12, 16]. On the contrary, RAS-mutated cancers tend to include follicular patterned tumors, e.g., follicular adenoma, follicular thyroid carcinoma, follicular variant PTC, and non-invasive follicular thyroid neoplasm with papillary-like nuclear features (NIFTP) [38, 39]. The majority of RAS mutant tumors show a less aggressive course and preserve thyroid differentiation. Because RAS mutations activate both MAPK and PI3K/AKT pathways, their detection may indicate lesions with intermediate malignant potential rather than overtly aggressive disease [30].

RET/PTC rearrangements and NTRK fusions are two further examples of important molecular changes in thyroid nodules. Fusion oncogenes result in constant kinase activity and MAPK pathway activation. RET/PTC rearrangements are prevalent in radiation-induced and childhood papillary thyroid cancers (PTCs), while NTRK fusions, which are rarer, have clinical significance in that they indicate sensitivity to TRK inhibitors. Consequently, detecting these fusion genes has assumed increasing importance both diagnostically and for therapy [38, 39]. PI3K/AKT pathway alterations such as PIK3CA gene mutations, AKT activation, and PTEN deletions occur more often in advanced cases of thyroid cancer, including dedifferentiated and poorly/anaplastic thyroid cancers. PTEN deletion and PI3K/AKT activation may also lead to treatment failure in terms of resistance to targeted drugs or radioactive iodine. Although less common in classic PTCs, PI3K/AKT pathway alterations may be clinically significant because of prognostic information derived from their presence [40].

More importantly, besides serving as a diagnostic tool, molecular changes are highly relevant in clinicopathologic behavior and current risk stratification systems. Tumors with BRAF V600E mutations, along with TERT promoter mutations, exhibit high recurrence rate, aggressive pathologic features, and poor disease-specific outcomes. On the other hand, RAS mutated tumors typically show low-risk features and a good prognosis. Molecular changes are now being integrated into ATA risk stratification systems and decision-making algorithms [35, 37]. Further advances in pathway crosstalk have elevated the importance of molecular diagnostics. Simultaneous activation of MAPK and PI3K/AKT pathways could help in identifying more aggressive tumors with high metastatic potential and resistance to therapy. This would enable identification of potential mechanisms of resistance and guide decisions on treatment combinations [4, 25]. There are several commercial platforms available for clinical utilization of molecular diagnostics in thyroid nodules. The most common of which include next-generation sequencing used in ThyroSeq v3 that tests for point mutations, gene fusions, copy number variations, and abnormal messenger RNA expression in relation to thyroid cancer. Another example is Afirma GSC that identifies messenger RNA expression patterns in order to differentiate between benign and malignant nodules [32, 37].

Molecular testing has helped avoid unnecessary thyroidectomy procedures through accurate preoperative risk stratification for malignancy. Moreover, molecular testing is now increasingly used to determine surgical extent, indications for radioiodine, follow-up, and candidacy for molecularly directed therapies [34]. Precision medicine techniques have gained ground, and this development has only increased the practical importance of molecular diagnostic tests. Discovery of actionable mutations like BRAF V600E, RET fusion genes, and NTRK gene rearrangements has led to the use of targeted therapies like BRAF inhibitors, RET inhibitors, and TRK inhibitors in cases of progressive or refractory metastatic disease. In addition, knowledge of adaptive pathway activation and mechanisms of drug resistance has led to exploration of combined therapies that target both MAPK and PI3K/AKT signaling pathways [36, 40].

In conclusion, molecular diagnostics has evolved into a crucial element in modern thyroid nodule workup and treatment of papillary thyroid cancer. Molecular diagnostics in combination with cytology, histopathology, imaging modalities, and clinical data provides clinicians with better tools to diagnose, characterize, plan therapy, and predict outcomes in papillary thyroid carcinoma patients.

### Clinicopathological correlations, prognostic implications and risk stratification

Genetic changes observed in papillary thyroid carcinoma (PTC) can be used to predict patient outcomes, guide treatment decisions, and determine long-term follow-up plans. Specific genetic changes are linked to certain patterns of pathology, biological properties, likelihood of recurrence, metastatic capacity, and sensitivity to RAI [31]. Of all genetic changes, BRAF V600E is the best understood prognostic marker for PTC. Many studies have shown that this mutation is correlated with more aggressive clinicopathological characteristics like extrathyroidal spread, lymph node involvement, advanced tumor stage, vascular invasion, disease recurrence, and lower rates of disease-free survival. Biologically, the BRAF mutation results in continuous MAPK activation, which downregulates genes responsible for thyroid differentiation, like SLC5A5 (NIS), thyroglobulin, and thyroid peroxidase, thus decreasing radioiodine uptake. The sustained ERK activation increases expression of proliferative, angiogenic, epithelial-mesenchymal transition, and apoptotic resistance genes [34].

It should be noted that the clinical value of BRAF V600E mutations increases considerably when combined with TERT promoter mutations, specifically those related to the alterations of the C228T and C250T positions. The combination results in a synergistic effect, increasing the expression of the TERT gene through ETS protein via the activated ERK pathway. Mutations of the two genes result in increased rates of recurrence, distant metastasis, dedifferentiation, tumor-specific death rate, and overall lower survival rate. The presence of these mutations can now be considered as a high-risk genotype for contemporary risk-stratification of thyroid cancer [31, 39].

Unlike the first category, RAS mutations usually show more benign behavior. Since RAS activates MAPK and PI3K/AKT pathways with lower intensity than BRAF V600E, RAS-mutated tumors usually preserve follicular morphology and some differentiation in thyroid tissue. In terms of clinicopathology, RAS mutations are mostly connected with encapsulated follicular variant PTCs and NIFTP, both characterized by low recurrence rate and good prognosis [21]. RET/PTC mutations tend to occur in children’s PTCs and radiation-associated PTCs. Although these mutations often manifest themselves with cervical lymph nodes metastasis, they remain sensitive to RAI treatment. Similarly, *NTRK* fusion-positive tumors often occur in younger patients and may demonstrate infiltrative growth patterns; however, they exhibit remarkable responsiveness to selective TRK inhibitors, significantly improving therapeutic outcomes in advanced disease [6, 25].

PI3K/AKT pathway mutations (such as PIK3CA mutations, AKT amplifications, PTEN deletions), however, are more commonly seen in patients with advanced disease, dedifferentiation, and transformation into poorly differentiated thyroid carcinomas or anaplastic thyroid carcinomas. The loss of PTEN and sustained activation of AKT signaling facilitate cancer cell survival, metabolism, invasion, and apoptosis resistance, promoting an aggressive tumor phenotype [40]. It should be noted that coactivation of the MAPK pathway and the PI3K/AKT pathway constitutes one of the critical determinants of aggressive tumor biology. Coactivation of pathways is associated with genomic instability, EMT, angiogenesis, immune escape, and resistance to targeted treatments and radioiodine therapy. Such tumors are prone to recur, metastasize, and have poor prognosis [21]. Molecular testing in combination with histologic and clinical features has greatly facilitated individualized risk stratification in PTCs. In contemporary ATA risk stratification criteria, the presence of molecular changes is increasingly considered in prognostication, thus guiding surgical and medical decision-making in terms of surgery extent, RAI application, surveillance frequency, and targeted treatment eligibility. Thus, molecular testing plays an indispensable role in precision medicine approaches in papillary thyroid carcinoma [17].

### Advances in targeted therapy

**Figure 2:**
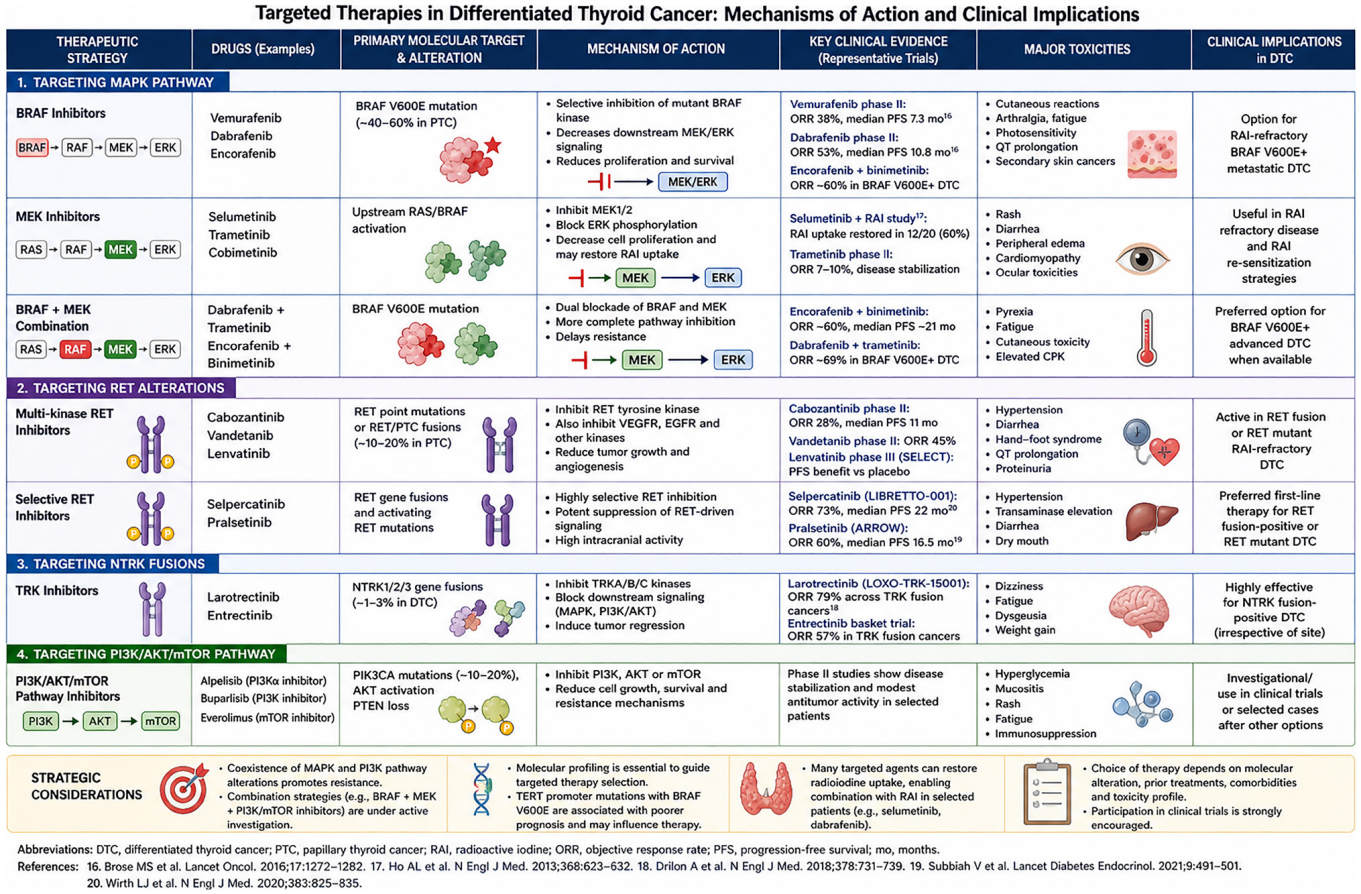
Targeted therapeutic strategies in papillary thyroid carcinoma and their mechanisms of action. Schematic representation of major approved and investigational therapies targeting MAPK and PI3K/AKT pathways in papillary thyroid carcinoma (PTC). BRAF inhibitors (vemurafenib, dabrafenib) and MEK inhibitors (trametinib, selumetinib) suppress MAPK signaling. RET inhibitors (selpercatinib, pralsetinib) and NTRK inhibitors (larotrectinib, entrectinib) target oncogenic fusion proteins. Multikinase inhibitors (lenvatinib, cabozantinib) inhibit angiogenesis and multiple receptor tyrosine kinases. PI3K/AKT/mTOR pathway inhibitors (buparlisib, everolimus) target survival and metabolic signaling. Mechanisms of therapeutic resistance include compensatory activation of parallel pathways, receptor upregulation, and feedback signaling loops.

Precision medicine in oncology has brought about a transformation in the treatment options for PTC with advanced and radioactive iodine refractory disease. Molecular profiling allows the development of customized cancer treatments that directly address molecular abnormalities in patients. Figure 2 provides a summary of approved and emerging molecular targeted therapies with their mechanisms of action.

#### Inhibitors of BRAF pathway

Inhibitors of BRAF pathway like Vemurafenib and Dabrafenib selectively inhibit mutant BRAF kinase and consequently downregulate the MAPK pathway by blocking MEK-ERK activity. They have shown good response in patients with BRAF V600E mutation thyroid carcinoma resistant to radioiodine therapy. Resistance has been noted to arise due to activation of alternative pathways such as RTK, RAS mutations, and PI3k/AKT.

#### Inhibitors of MEK pathway

Inhibitors of MEK pathway like Trametinib and Selumetinib can be used to target MAPK pathway and may induce NIS expression and radioiodine avidity.

#### Inhibitors of RET

Selectivity of RET inhibitors such as Selpercatinib and Pralsetinib makes it possible to achieve excellent clinical outcomes with fewer toxicities among patients with RET fusion-positive thyroid cancer.

#### TRK inhibitors

The TRK inhibitors Larotrectinib and Entrectinib are highly selective for NTRK gene fusions. These drugs exhibit high activity in solid tumors that harbor TRK rearrangements including thyroid cancers.

#### Inhibitors of PI3K/AKT/mTOR signaling pathway

The mTOR inhibitors Everolimus and the PI3K inhibitor Buparlisib are currently being investigated in the treatment of advanced thyroid cancer characterized by PI3K signaling activation. Despite the lack of consistency in clinical results, combined treatment of MAPK and PI3K signaling pathways is gradually becoming the subject of interest.

#### Multikinase inhibitors

Lenvatinib and Cabozantinib are multikinase inhibitors that affect VEGFR, FGFR, RET, and MET signaling cascades, contributing to the antiangiogenic effect and inhibiting the growth of the tumor tissue. Currently, these inhibitors are approved for use in advanced RAI-refractory differentiated thyroid carcinoma. Combined treatment with BRAF, MEK, PI3K, or mTOR inhibitors can provide additional benefits by addressing pathway cross-talk and drug resistance. Table 1 provides an overview of approved and experimental therapies used in thyroid carcinoma patients, along with their molecular target and mechanism of action.

**Table 1:** Targeted therapies in papillary thyroid carcinoma

Therapeutic agent	Molecular target	Mechanism of action	Clinical status in PTC
Vemurafenib	BRAF V600E	Selective inhibition of mutant BRAF kinase leading to suppression of MAPK signaling	Investigational/RAI-refractory PTC
Dabrafenib	BRAF V600E	Inhibits mutant BRAF kinase; reduces downstream MEK/ERK signaling	Approved (in combination for BRAF-mutant ATC)
Trametinib	MEK1/2	Inhibits MEK downstream of BRAF, blocking ERK activation	Combination therapy
Selumetinib	MEK1/2	Enhances NIS expression and restores radioiodine uptake	Investigational
Selpercatinib	RET fusion	Highly selective RET kinase inhibition	FDA-approved
Pralsetinib	RET fusion	Selective RET inhibition with potent anti-tumor activity	FDA-approved
Larotrectinib	NTRK fusion	Selective TRK inhibition	FDA-approved (tumor-agnostic)
Entrectinib	NTRK fusion	Pan-TRK, ROS1, ALK inhibition	FDA-approved (tumor-agnostic)
Everolimus	mTOR	Inhibits mTOR signaling downstream of PI3K/AKT	Investigational
Buparlisib	PI3K	Pan-PI3K inhibition suppressing AKT signaling	Investigational
Cabozantinib	VEGFR/MET/RET	Multikinase inhibitor targeting angiogenesis and tumor growth	FDA-approved (RAI-refractory DTC)
Lenvatinib	VEGFR/FGFR/PDGFR	Antiangiogenic multikinase inhibitor	FDA-approved (RAI-refractory DTC)

## CONCLUSIONS

Aberrant activation of MAPK and PI3K/AKT pathways is considered to be one of the major mechanisms underlying papillary thyroid carcinoma. Although the role of MAPK pathway abnormalities in carcinogenesis seems to be more important, the PI3K/AKT pathway activation is considered to be responsible for tumor progression, undifferentiated growth, and acquired resistance. Molecular interaction of both pathways increases oncogenicity and therapy resistance.

Development of novel molecular diagnostics tests and targeted therapies helped to enhance personalized treatment approaches in papillary thyroid carcinoma patients. The future might hold new approaches in combinational therapy of aggressive and therapy-resistant forms of papillary thyroid carcinoma.

## Abbreviations

AKT: Protein Kinase B
ATC: Anaplastic Thyroid Carcinoma
AUS/FLUS: Atypia of Undetermined Significance/Follicular Lesion of Undetermined Significance
BRAF: v-Raf Murine Sarcoma Viral Oncogene Homolog B
CCDC6: Coiled-Coil Domain Containing 6
EGFR: Epidermal Growth Factor Receptor
EFVPTC: Encapsulated Follicular Variant of Papillary Thyroid Carcinoma
ERK: Extracellular Signal-Regulated Kinase
FN/SFN: Follicular Neoplasm/Suspicious for Follicular Neoplasm
GSC: Genomic Sequencing Classifier
HRAS/NRAS/KRAS: Harvey/Neuroblastoma/Kirsten Rat Sarcoma Viral Oncogene
